# Inhibition of Digestive Enzyme and Stimulation of Human Liver Cells (HepG2) Glucose Uptake by Date Seeds Extract

**DOI:** 10.1155/2020/4290702

**Published:** 2020-07-29

**Authors:** Hira Shakoor, Fatima Abdelfattah, Khaula Albadi, Mentalla Adib, Jaleel Kizhakkayil, Carine Platat

**Affiliations:** Department of Food, Nutrition and Health, College of Food and Agriculture, United Arab Emirates University, P.O. Box 15551, Al Ain, UAE

## Abstract

Type 2 diabetes mellitus is increasing worldwide, and the United Arab Emirates is presenting one of the world's highest prevalence rates. Dietary polyphenols exert an antidiabetic effect by modulating carbohydrates digestion and cellular glucose uptake. Due to their particularly high content in polyphenols, date seeds represent a potential antidiabetic agent. This study aims to determine if date seed polyphenols inhibit the activity of the enzymes (*α*-amylase and *α*-glucosidase), responsible for the digestion of carbohydrates and modulating the glucose uptake by human liver cells. *In vitro* activity of the intestinal *α*-glucosidase, pancreatic *α*-amylase, the glucose uptake by HepG2 cells, and the expression of GLUT4 and AMPK analyzed by western blotting (with and without date seeds extract). Our result showed that the maximum enzymes inhibition was obtained with 400 *μ*g/mL and 900 *μ*g/mL DSE for *α*-amylase and *α*-glucosidase, respectively. The HepG2 cell viability significantly decreased up to 80% at 4000 *μ*g/mL DSE. The expression of GLUT4 was higher at 100 *μ*g/mL DSE (with insulin and without insulin). However, the expressions of P-AMPK and AMPK were increased by DSE, mainly in a non-insulin-dependent manner. Therefore, DSE, by inhibiting carbohydrate digestion and stimulating glucose uptake by HepG2, can potentially demonstrate the therapeutic potential for diabetes management.

## 1. Introduction

Type 2 diabetes mellitus (T2DM) is a complex metabolic disorder characterized by hyperglycemia resulting from abnormal glucose metabolism. It is associated with many complications, including hypertension, retinopathy, nephropathy, and neuropathy [1]. T2DM is now among the top ten causes of death in the world [2]. It is continuously increasing despite significant therapeutic advancement. Globally, 425 million individuals are suffering from T2DM, which may increase to 693 million by 2045 [2]. In the United Arab Emirates (UAE), the prevalence of T2DM is 18.7%, one of the greatest in the world and expected to reach 21.4% by 2030 [3]. This trend adds a tremendous burden to societies and healthcare systems. Hence, it is urgent to identify new strategies to stop this progression.

Postprandial hyperglycemia is an independent risk factor for T2DM regulated in two ways: the intestinal absorption of glucose, which depends on the breaking down of carbohydrates by enzymes including *α*-glucosidase and *α*-amylase, and the cellular uptake of glucose [4]. The liver plays a central role in the regulation of glucose homeostasis either by storing glucose as glycogen or releasing it into the blood after intestinal absorption. Further, the liver is the place where glycogen breakdown, glycolysis, and neoglucogenesis can occur [5]. Nowadays, many drugs are available for T2DM that can help decrease blood glucose levels, but those drugs have many side effects. Therefore, the therapeutic approach should be taken into consideration for the management of T2DM [6]. Interestingly, diet and a healthy lifestyle have a significant role in the prevention of T2DM. Diverse underlying mechanisms have been identified, including retarding the absorption of glucose by inhibiting carbohydrates hydrolyzing enzymes (*α*-amylase and *α*-glucosidase) and increasing the expression of glucose transporters in various human cells [7]. Dietary compounds likely to exert such effects would represent a promising alternative to drugs in the prevention and treatment of T2DM.

Polyphenols are a large and heterogeneous group of phytochemicals (plant-based food) such as flavonoids, phenolic acids, lignans, and stilbenes [8, 9]. Several hundred different polyphenols have been identified in vegetables, fruits, and cereals [10, 11]. Admittedly, polyphenols reported having many beneficial health properties such as antioxidant, antibacterial, antiviral, anti-inflammatory, and anticancerous [12]. Polyphenols are known to exhibit antidiabetic properties due to their ability to influence glucose metabolism. For instance, polyphenols have been shown to modulate digestive enzymes involved in carbohydrate digestion, stimulate insulin secretion by *β*−cells, activate insulin receptors, and regulate glycemia by stimulating glucose uptake in insulin-sensitive tissue and by modulating hepatic glucose output [11–13]. Such properties have been reported for polyphenols from coffee, guava, tea, whortleberry, olive oil, propolis, chocolate, red wine, grape seed, and cocoa [14]. Remarkably, polyphenols can inhibit digestive enzymes like *α*-amylase and *α*-glucosidase, leading to a reduced release of glucose after a meal [15, 16]. In addition, polyphenols can modulate glucose transportation by stimulating GLUT transporters, among which the insulin-sensitive GLUT4 is notable. Polyphenols have been involved in promoting GLUT4 translocation in muscles and adipose tissues [17, 18]. Phenolic compounds could also restore the phosphorylated level of AMPK (P-AMPK) in hepatic cells to maintain glucose homeostasis [19, 20]. Activation of AMPK in the liver, skeleton muscle, and adipose tissue helps to promote glucose uptake, insulin sensitivity, and fatty acid oxidation [21, 22]. Consequently, any plant or plant-derived food rich in polyphenols present a potential for the prevention and the management of T2DM.

Date seeds are a widely available by-product in the Middle East region. There are about 40 million date trees in the UAE, and the country is one of the largest producers of dates in the world [23]. Interestingly, date seeds are particularly rich in polyphenols, with a content higher than other famous polyphenol-rich food products like grape or tea [24–27]. Depending on the variety, a concentration in polyphenols between 1864.82 and 4768.87 mg GAE/100 g was reported [26]. A detailed exploration of the polyphenolic compounds identified flavan-3-ols, especially catechins and epicatechins, as the most abundant in date seeds, with up to 50.18 g/kg flavan-3-ols in the Khalas variety [24, 25]. A detailed qualitative and quantitative identification of polyphenols in date seeds was performed using HPLC-mass spectrometry and found abundant amounts of flavan-3-ols, phenolic acids, flavones, and flavonols [28]. *In vitro* and *in vivo* studies also illustrated the antioxidant property in date seeds [29–31]. However, the possible effect of date seeds on glucose homeostasis still remained unknown.

Therefore, our study's primary purpose was to determine if date seeds extract (DSE) could contribute to glucose homeostasis by modulating glucose intestinal digestion and glucose uptake by human liver cells and, then, to investigate some potential mechanisms underlying these possible effects.

## 2. Materials and Methods

### 2.1. Date Seed Extract (DSE) Preparation

Date palm (*Phoenix dactylifera* L.) seeds of the Khalas variety were used in this trial. The Al Foah, Company-Emirates Dates factory, provided date seeds. Dates were collected randomly from tamr (fully ripe dates) batches at the end of the season, with no preference to size, color, appearance, or firmness.

Date seeds powder was prepared by milling cleaned and dried date seeds. The powder was sieved by BZS 200 sieve machine and particles of less than 300 microns were used for the study as powder. DSE was prepared by extracting the powder with ethanol : water (1 : 1) solution. The extract was filtered using Whatman filter paper, was reduced under nitrogen, and was vacuum dried to yield the extract. The detailed polyphenolic contents of DSE have already been described in one of our previous works [28].

### 2.2. Carbohydrate Digestion Inhibition

#### 2.2.1. *α*-Amylase Inhibitory Assay

The assay was carried out using a modified procedure version of McCue and Shetty, 2004 [32]. A total of 250 *μ*L of date seed extract (0–1500 *μ*g/mL) was placed in a tube and 250 *μ*L of 0.02 M sodium phosphate buffer (pH 6.9) containing *α*-amylase solution (0.5 mg/mL) was added. The tubes' content was preincubated at 25°C for 10 mins, after which 250 *μ*L of 1% starch solution in a 0.02 M sodium phosphate buffer (pH 6.9) was added timed intervals. The reaction mixtures were incubated at 25°C for 10 min. The reaction was stopped by adding 500 *μ*L of dinitrosalicylic acid (DNS) reagent and then incubated in boiling water for 5 min and cooled to room temperature. The content of each test tube was diluted with 5 mL of distilled water. The absorbance was measured at 540 nm in a spectrophotometer (Spectrumlab S23A, Globe Medical, England). The control was prepared in the same except that the extract was replaced with distilled water. The *α*-amylase inhibitory activity was calculated as in the following equation:

(1) $$\begin{array}{c} \%\text{inhibition}=\left\{\frac{\left(\text{Ac}-\text{Ae}\right)}{\text{Ac}}\right\}100, \end{array}$$

where Ac and Ae are the absorbance of the control and extract, respectively.

#### 2.2.2. *α*-Glucosidase Inhibitory Assay

The *α*-glucosidase activity on the extract was determined according to the method described by Kim et al. using *α*-glucosidase from *Saccharomyces cerevisiae* [33]. The substrate solution p-nitrophenyl glucopyranoside (pNPG) (3.0 mM) was prepared in 20 mM phosphate buffer, pH 6.9. 100 *μ*L of *α*-glucosidase (1.0 U/mL) was preincubated with 50 *μ*L of the different concentrations of the extracts (0–4000 *μ*g/mL) for 10 mins. Then 50 *μ*L of 3.0 mM (pNPG) as a substrate dissolved in 20 mM phosphate buffer (pH 6.9) was added to start the reaction. The reaction mixture was incubated at 37°C for 20 mins and stopped by adding 2 mL of 0.1 M Na_2_CO_3_. The *α*-glucosidase activity was determined by measuring the yellow-colored para-nitrophenol released from pNG at 405 nm. The results (% inhibition) are expressed as a percentage of the blank (control) as in the following equation.

(2) $$\begin{array}{c} \%\text{inhibition}=\left\{\frac{\left(\text{Ac}-\text{Ae}\right)}{\text{Ac}}\right\}100, \end{array}$$

where Ac and Ae are the absorbance of the control and extract, respectively.

### 2.3. HepG2 Liver Cells Culture

Culture in 75 cm^2^ cell culture flasks, at 37°C, in a humidified atmosphere of 5% CO_2_/95% O_2_, at a seeding density of approximately 105 cells/cm^2^, in Eagle's minimal essential medium, was supplemented with 10% v/v heat-inactivated FBS and 100 U/mL penicillin-streptomycin. Cells grow until 80–90% confluence. Cells were used up to passage 20 for the experiments.

### 2.4. Cell Viability Test

HepG2 cells were cultured in the 96 wells plate (1 × 10^4^ cells/well) and incubated overnight. After 24 hours, they were treated with various concentrations (ranging from 0 to 4000 *μ*g/mL) of date seeds extract for 24 hours. 10 *μ*L of WST-1(4-[3-(4-Iodophenyl)-2-(4-nitro-phenyl)-2H-5-tetrazolio]-1,3-benzene sulfonate) solution was added to each well and incubated further for 4 hours. The absorbance was measured with a spectrophotometer at 420 nm by using a microplate reader.

### 2.5. Cell Glucose Uptake

Glucose uptake was assayed according to the established protocol from a commercial glucose uptake kit (ab136955; Abcam). In brief, HepG2 cells were seeded (1 × 10^5^ cells/well) in a 24-well plate overnight. After 24 hours, media was replaced with serum-free DMEM/F12 medium and incubated overnight. Krebs-Ringer Phosphate-Hepes buffer was added with an incubation time of 40 minutes. Subsequently, cells were stimulated with 20, 40, and 100 *μ*g/mL date seeds extract for 4 hours and insulin (1 *μ*M) (Sigma Aldrich, USA) for 15 minutes. Then, 10 mM 2-deoxyglucose was added and incubated for an additional 20 min. Cells were washed three times with cold PBS and lysed with extraction buffer, then frozen at −80°C for 10 min, and heated at 85°C for 40 min. After cooling on ice for 5 min, the lysates were neutralized by adding neutralization buffer. Centrifugation was done, and the remaining lysate was diluted with assay buffer. Finally, the end product was produced with an amplification step as per the kit protocol. Absorbance was measured at 412 nm by using a multiscan microplate reader.

### 2.6. GLUT4 and AMPK Protein Expression

HepG2 cells were seeded (1 × 10^5^ cells/well) in a 24-well plate overnight and next day treated with DSE (40 and 100 *μ*M) without insulin (1 *μ*M) for 4 hours. HepG2 cells were washed with phosphate buffer saline (PBS). 200 *μ*L of RIPA Cell lysis buffer (50 mM Tris-HCl pH 7.4, 150 mM NaCl, 1 mM ethylenediaminetetraacetic acid, 1% Triton X100, 0.1% sodium dodecyl sulphate (SDS), 10 mM NaF, 1 mM Na_3_VO_4_, and 50 mM Na_4_P_2_O_7_) containing 1% protease inhibitor cocktail, 1 mm phenyl methyl sulfonyl fluoride (PMSF), and 10 mm dithiothreitol (DTT) was added to the cells. The cell lysates were centrifuged at 14,000 rpm for 15 min at 4°C. Total protein was determined by Bio-Rad protein assay, diluted with 6 × loading buffer, and boiled at 100°C for 5 minutes. Loaded 40 *μ*g/lane of proteins samples, separated by SDS–PAGE, was transferred onto a nitrocellulose membrane by wet transfer using a Bio-Rad Electrotransfer apparatus. The membranes were blocked with milk for 1 hour at room temperature and immunoblotted using polyclonal primary antibodies against GLUT4, P-AMPK, AMPK, and *ß*-actin antibodies (Cell signaling, USA). The membranes were incubated for 2 hours with primary antibodies and then with appropriate horseradish peroxidase-conjugated secondary antibodies for 1 hour. The band densities were detected by detecting the intensity of the band and using an enhanced chemiluminescence detection kit (Thermo, USA). The band densities were quantified using an image analyzer Quantity One System (Bio-Rad).

### 2.7. Statistical Analysis

**Figure 1:**
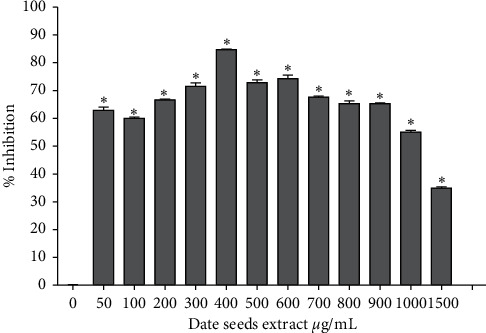
Effect of date seeds extract (0–1500 *μ*g/mL) on *α*-amylase activity ANOVA was used to compare between control (0 *μ*g/mL date seeds extract) and other date seeds extract concentrations. Values are the mean ± SD calculated from three independent experiments. ^*∗*^Significantly (<0.05) different from 0 *μ*g/mL date seeds extract.

**Figure 2:**
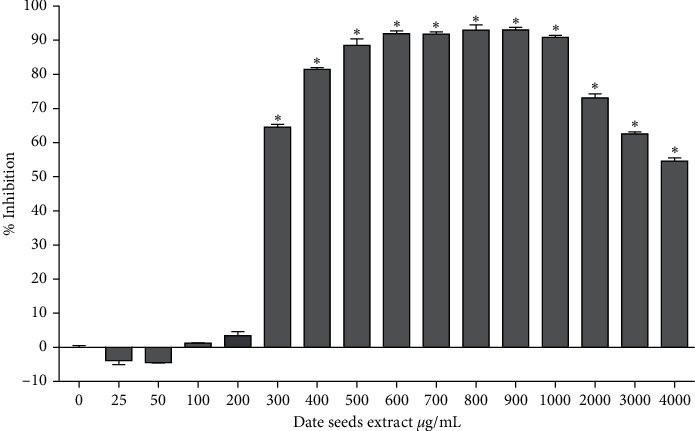
Effect of date seeds extract (0–4000 *μ*g/mL) on *α*-glucosidase activity ANOVA was used to compare between control (0 *μ*g/mL date seeds extract) and other date seeds extract concentrations. Values are the mean ± SD calculated from three independent experiments. ^*∗*^Significantly (<0.05) different from 0 *μ*g/mL date seeds extract.

**Figure 3:**
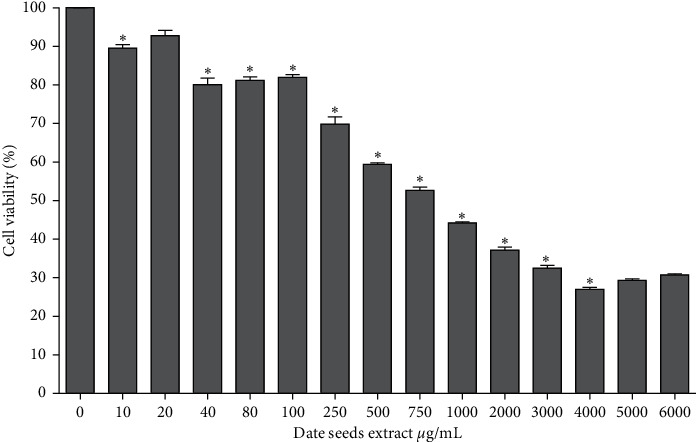
Effect of date seeds extract on HepG2 cell viability ANOVA was used to compare between control (0 *μ*g/mL date seeds extract) and other date seeds extract concentrations. Values are the mean ± SD calculated from three independent experiments. ^*∗*^Significantly (<0.05) different from 0 *μ*g/mL date seeds extract.

**Figure 4:**
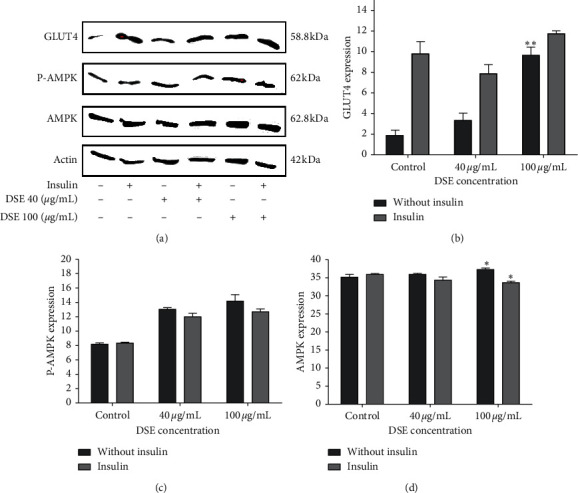
Effect of date seeds extract on the level of expression of GLUT4, AMPK, and P-AMPK proteins in human liver cells HepG2, with or without date seeds extract, in presence or not of insulin ANOVA was used to compare between control (0 *μ*g/mL date seeds extract, with and without insulin) and other date seeds extract concentrations. Values are the mean ± SD. ^*∗*^Significantly (<0.05), ^*∗∗*^Highly significant (<0.01) difference from 0 *μ*g/mL date seeds extract.

**Figure 5:**
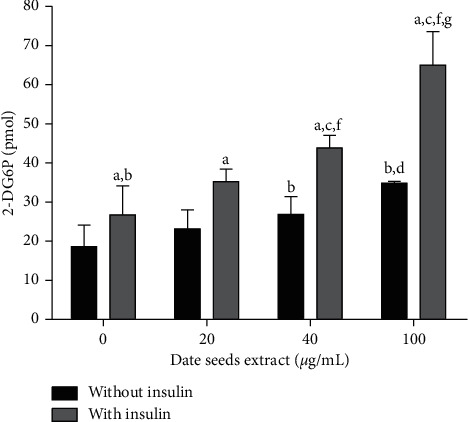
Effect of date seeds extract (0, 20, 40, and 100 *μ*g/mL) on glucose uptake by human liver cells HepG2. Values are the mean ± SD calculated from three independent experiments. ^a^Statistically significant difference with the same condition without insulin. ^b^Statistically significant difference with the control without insulin. ^c^Statistically significant difference with the control with insulin. ^d^Statistically significant difference with DSE 20 *μ*g/mL without insulin. ^e^Statistically significant difference with DSE 40 *μ*g/mL without insulin. ^f^Statistically significant difference with DSE 20 *μ*g/mL with insulin. ^g^Statistically significant difference with DSE 40 *μ*g/mL with insulin.

Statistical analyses were performed by using the SPSS software v.25. Means ± SD or % were calculated, as appropriate. Experiments were done at least in triplicate; then the average was calculated. The statistical significance of experimental observations was determined using ANOVA followed by Dunnett (Figures 1–4(c)) and Tukey's posttest (Figure 5). Statistical significance was set at *p* < 0.05.

## 3. Results and Discussion

Briefly, our data showed that DSE inhibited *α*-amylase and *α*-glucosidase and increased the expression of GLUT4, AMPK, and P-AMPK. These results support a potential role of DSE in the regulation of glucose homeostasis. These beneficial effects of DSE may be related to its high polyphenolic contents, especially the abundance of phenolics and flavan-3-ols for which antidiabetic properties have already been reported [25].

### 3.1. Polyphenolic Contents of DSE

Hilary et al. [28] characterized polyphenols from Khalas variety date seeds in three different forms: date seed pita bread (DSB), date seed powder (DSP), and date seed extract (DSE). The main compounds detected in all the three forms of date seeds were hydroxycinnamic acids, flavonols, flavanols, flavones, and hydroxybenzoic acids, which are mentioned in (Table 1).

### 3.2. Inhibition of *α*-amylase and *α*-glucosidase Activity

The effect of DSE on glucose digestion was investigated *in vitro* by measuring the activity of the enzymes: *α*-amylase and *α*-glucosidase (with or without DSE). A significant percentage of inhibition of *α*-amylase, compared to the control, was observed at different concentrations of DSE, starting from 50 *μ*g/mL and up to 1500 *μ*g/mL (Figure 1). The maximum level of inhibition of *α*-amylase (84.68%) was detected with 400 *μ*g/mL DSE. Similarly, *α*-glucosidase was inhibited by DSE (Figure 2), from 300 *μ*g/mL to 4000 *μ*g/mL DSE. The maximum level of inhibition *α*-glucosidase (93.04%) was detected at 900 *μ*g/mL DSE.

The two digestive enzymes, *α*-amylase and *α*-glucosidase, are involved in the breakdown of starch (polysaccharide, oligosaccharide) into simpler substances like disaccharides and monosaccharides. Inhibiting these enzymes hinders starches catabolism, hence, digestion and absorption of carbohydrates, thereby reducing postprandial glucose concentration [34, 35]. Evidence showed that other polyphenol-rich products like tea, rosemary, pears, cocoa, lentils, and berries could inhibit *α*-amylase and *α*-glucosidase activity [36–38]. It was found that 0.05 mg/mL of tea polyphenols was reported to inhibit 61% *α*-amylase and *α*-glucosidase in vivo and in vitro conditions [39]. The maximum level of inhibition with DSE in our findings was 84.68% for *α*-amylase and 93.04% *α*-glucosidase, which is higher than tea polyphenols in previous studies. Polyphenols like anthocyanins, cyanidin 3-arabinoside, caffeic acid, rosmarinic acid, resveratrol, catechol, and protocatechuic acid would possess the most potent activity against digestive enzymes [34, 37, 40, 41]. Interestingly, many of these compounds, including protocatechuic acid, are abundant in DSE, suggesting that the inhibitory effect of DSE on digestive enzymes could be attributed to their polyphenols.

### 3.3. Viability of HepG2 Cell

The potential cytotoxic effect of DSE was investigated, determining their impact on the viability of a human hepatoma cell line, HepG2. The HepG2 cell death rate was dose-dependent and about 20% of cell death was observed 40 *μ*g/mL after the treatment with DSE for 24 hours of incubation. As the concentration of DSE is increasing, the inhibition of HepG2 cell proliferation increased significantly, especially from 750 *μ*g/mL to 4000 *μ*g/mL. Maximum inhibition of about 80% was observed around 4000 *μ*g/mL of DSE (Figure 3). Similarly, other studies showed that phenolic compounds such as flavonol, tannin, and anthocyanin inhibit the proliferation of HepG2 cells [42, 43]. Our result is similar to the findings of Yi et al., which reported 50% inhibition of HepG2 cell population growth at 70 *μ*g/mL of anthocyanin fractions [43]. Moreover, another study illustrated that food-derived phenolic content like chlorogenic acid and epicatechin reduced cell viability 15–20% after 18 hours of treatment [44].

### 3.4. Glucose Uptake by HepG2 Cells

The effect of DSE on glucose uptake by HepG2 cells was analyzed at a concentration of 20, 40, and 100 *μ*g/mL. Glucose uptake assay cells were exposed to the extract for only 4 hours and there was no influence on the cell viability with this concentration. So, these concentrations were not enough to make any changes in free glucose variation with cell death and ideal for the glucose uptake experiments. Glucose uptake by HepG2 cells (Figure 5) was significantly raised with DSE at 40 *μ*g/mL and 100 *μ*g/mL compared to the control without insulin. Similarly, in the presence of insulin, glucose uptake was increased with DSE at 40 *μ*g/mL and even more at 100 *μ*g/mL, compared to the control with insulin. These effects were higher compared to the same concentrations without insulin. Our results agree with other studies on natural rich polyphenols ingredients like berries extract, showing that polyphenols like anthocyanins and proanthocyanidins stimulate glucose uptake by liver and muscle cells concentrations ranging from 0.1 to 10 *μ*M [45–47]. Beyond this, our results indicated that DSE could modulate the glucose uptake by HepG2 cells via both insulin and non-insulin-dependent pathways. It is known that dietary polyphenols influence peripheral glucose uptake in both insulin-sensitive and non-insulin-sensitive tissues [48–51]. *In vitro* studies showed that some polyphenolic compounds, including quercetin, resveratrol, epigallocatechin-3-gallate, and procyanidin, helped to improve insulin-dependent glucose uptake in muscle cells and adipocytes by translocation of glucose transporter, GLUT4, to plasma membrane mainly through induction of the AMP-activated protein kinase (AMPK) pathway [52–56].

### 3.5. Level of Expression of GLUT4, AMPK, and P-AMPK

The level of expression of the proteins GLUT4, AMPK, and P-AMPK was assessed by Western Blotting, for 40 *μ*g/mL and 100 *μ*g/mL DSE (Figure 4). As per the density of the bands (Figures 4(a)–4(c)), DSE significantly stimulated the expression of GLUT4 protein at 100 *μ*g/mL without insulin. However, with insulin, the expression of GLUT4 also increased but did not reach statistical significance. Similarly, AMPK expression increased significantly at 100 *μ*g/mL (with and without insulin). However, expression of P-AMPK protein increased with DSE in a dose-dependent manner and, to a greater extent, in the absence of insulin.

The translocation of GLUT4 is essential to maintain glucose homeostasis [57, 58]. GLUT4 translocation and AMPK phosphorylation increase insulin sensitivity, reduce insulin resistance, and prevent hyperglycemia with the help of polyphenols [55]. GLUT4 and AMPK were related by a mechanical pathway starting with the activation of AMPK by polyphenols and resulting in the induction of GLUT4 transporters translocation [52, 59–61]. The addition of insulin enhanced the effect of DSE on GLUT 4 level but not on AMPK level, which indicated that DSE could also translocate GLUT4 via the direct insulin-dependent mechanism, inducing the translocation of GLUT4 transporters [51, 62, 63]. AMPK is well-known to work as an energy sensor [64, 65]. In the case of energy demand, AMPK is activated, promoting glucose uptake, glycolysis, and fat oxidation [66]. Therefore, DSE because of it phenolic content could help in controling blood glucose concentration, thus, preventing T2DM by promoting glucose/fat catabolism and transport.

### 3.6. Limitations

This study is presenting some limitations. Here, the expression of GLUT4 was only measured, whereas, the effect of DSE on the expression of other transporters like GLUT1 (can be expressed to the same extent in cells and especially in liver cells) and GLUT 2 (primary glucose transporter in the liver) need to be analyzed. The overall modulation of glucose uptake will depend on the effect on each type of transporters. However, in adipose tissue, GLUT1 is expressed along with GLUT4 [67]. So, the translocation of these transporters is essential for the reduction of plasma glucose levels.

Similarly, among all digestive enzymes, only two were considered, that is, *α*-amylase and *α*-glucosidase because they are involved in carbohydrate digestion. However, inhibition of lipase activity is also crucial for controling obesity [68], which is a risk factor for diabetes. So, measuring the effects of date seed polyphenols on lipase inhibition will give us future direction.

Besides, after oral ingestion, polyphenols are likely to be transformed in the digestive tract and interact with the gut microbiota so that polyphenols in the blood circulation may differ significantly from the natural polyphenols, which is still unknown. However, in a recent human study, the metabolites of DSE polyphenols in urine were described, after oral ingestion [31]. It was reported that, in the first three hours of DSE intake, there was a significant increase in levels of aromatic acids metabolites such as protocatechuic acid, hydroxybenzoic acid, vanillic acid, vanillic acid sulphate, and ferulic acid sulphate as compared to the baseline. Seed polyphenols and metabolites have antioxidant effect because they upregulate enzymatic defence system, that is, GSH, and decrease oxidative stress [31].

## 4. Conclusions

In conclusion, our data highlighted the inhibitory effect of DSE on the activity of *α*-amylase and *α*-glucosidase, both human digestive enzymes responsible for the breakdown of dietary carbohydrates. Further, it was shown that DSE was able to stimulate the translocation of GLUT4 and increased the expression of energy sensor AMPK and P-AMPK, in cells from the liver (central organ for glucose and fat metabolism). It was also observed that phenolic compounds in DSE could inhibit HepG2 cell proliferation, knowing that DSE is relatively inexpensive and readily available [31]. DSE could be a possible alternative for the management of type 2 diabetes. Further research needs to be done to confirm the therapeutic potential of date seed polyphenols.

## Figures and Tables

**Table 1:** Polyphenols in different forms of date seeds.

Polyphenols present in date seeds	Compounds detected	Forms of date seeds
Hydroxycinnamic acids	Caffeic acid Caffeic acid hexoside	*DSE*
	N1,N8-dicaffeoyl spermidine Caffeoylshikimic acid	*DSP, DSE, DSB*
	N1,N4-dicaffeoyl spermidine	*DSP and DSE*

Flavonols	Quercetin Quercetin hexoside Quercetin hexoside sulphate Kaempferol hexoside	*DSE, DSP*

Flavanols	Procyanidin	*DSE, DSB*
	Catechin Epicatechin	*DSP, DSE, DSB*

Flavones	Diosmetin-7-o-rutinoside 7-O-hexosyl diosmetin Diosmetin hexoside sulphate	*DSP, DSE*

Hydroxybenzoic acids	Protocatechuic acid *p*-hydroxybenzoic acid Syringic acid hexoside	*DSP, DSB, DSE*

DSP: date seeds powder, DSE: date seeds extract, and DSB: date seeds bread.

## Data Availability

The data, in excel format and anonymous, to protect participants' privacy, are available upon request to the corresponding author.
